# A Prospective Stratified Case-Cohort Study on Statins and Multiple Adverse Events in Japan

**DOI:** 10.1371/journal.pone.0096919

**Published:** 2014-05-08

**Authors:** Nobuhiro Ooba, Tsugumichi Sato, Akira Wakana, Takao Orii, Masaki Kitamura, Akira Kokan, Hideaki Kurata, Yoshihiro Shimodozono, Kenichi Matsui, Hiroshi Yoshida, Takuhiro Yamaguchi, Shigeru Kageyama, Kiyoshi Kubota

**Affiliations:** 1 Department of Pharmacoepidemiology, Graduate School of Medicine, University of Tokyo, Tokyo, Japan; 2 Department of Pharmacy, Faculty of Pharmaceutical Sciences, Tokyo University of Science, Chiba, Japan; 3 Drug Safety Research Unit Japan, Tokyo, Japan; 4 MSD K.K., Tokyo, Japan; 5 NTT Medical Center Tokyo, Tokyo, Japan; 6 Department of Pharmacy, The Jikei University School of Medicine, Tokyo, Japan; 7 EliLilly Japan, K.K., Hyogo, Japan; 8 Division of Diabetes, Metabolism and Endocrinology, Department of Internal Medicine, The Jikei University School of Medicine, Tokyo, Japan; 9 Department of Clinical Pharmacy and Pharmacology, Graduate School of Medical and Dental Sciences, Kagoshima University, Kagoshima, Japan; 10 Office for Promoting Medical Research, Showa University, Tokyo, Japan; 11 Department of Laboratory Medicine, The Jikei University Kashiwa Hospital, Chiba, Japan; 12 Department of Public Health and Forensic Medicine, Tohoku University Graduate School of Medicine, Miyagi, Japan; 13 Division of Clinical Pharmacology and Therapeutics, The Jikei University School of Medicine, Tokyo, Japan; Medical University Innsbruck, Austria

## Abstract

**Purpose:**

To assess the association between statins and diverse adverse events in Japanese population.

**Methods:**

New users of statin who started statin after 6-month period of non-use were identified in 68 hospitals between January 2008 and July 2010. In addition to the random sample subcohort, we selected additional subcohort members to make the stratified sample subcohort have at least one patient in all subgroups stratified by each combination of statin and hospital. By abstraction from medical records, detailed information was obtained for all potential cases and pre-selected subcohort members. The event review committee consisting of 3 specialists judged whether possible cases met the definition of one of the adverse events of interest, and for adjudicated cases the committee further judged whether statin was a certain, probable or possible cause of the occurrence of the event. Adjusted for covariates including age, gender, status of “switcher”, use of high daily dose and comorbidities at baseline, hazard ratio (HR) was estimated by the Cox proportional hazards model with Barlow’s weighting method. Data were also analyzed by the method proposed by Breslow in 2009.

**Results:**

A total of 6,877 new users of a statin were identified (median age: 66 years; males: 52%). The hazard ratios of increase in serum creatinine for atorvastatin and fluvastatin have wide confidence intervals, but both of the point estimates were around 2.5. Estimates of hazard ratios by the method of Barlow (1999) were similar to those by the method of Breslow (2009).

**Conclusions:**

Use of statin was not associated with a significant increased risk for renal, liver and muscle events. However, the hazard ratio of increase in serum creatinine tended to be high with atorvastatin and fluvastatin to require further studies.

## Introduction

Statins are widely used for the treatment of dyslipidemia to prevent cardiovascular diseases. In Japan, heart and cerebrovascular diseases are the second and third causes of mortality [1] and the number of patients with hyperlipidemia is estimated as 1.43 million in 2008 [2]. However, statins have major adverse effects on liver and muscles including rhabdomyolysis [3]. Renal toxicity was recognized as a concern associated with the use of rosuvastatin [4], [5] and further addressed recently [6]–[8]. Renal toxicity may be augmented with high dose and the maximum daily dose of rosuvastatin was set as 20 mg/day in Japan (half of 40 mg/day in the US and EU) based on a pharmacokinetic study of a small number of patients [9]. A company-sponsored post-marketing drug use investigation (DUI) [10] was conducted for rosuvastatin between 2005 and 2006. As in most DUIs conducted under the legislation for re-examination of new drugs, the DUI for rosuvastatin did not have a comparator [10]. In this DUI where the maximum daily dose was 5 mg/day or less for 98% of patients, the increase in serum creatinine by over 30% from the baseline was observed in 350 (4.1%) of 8,553 patients during the 12-week observation period [11]. Though the incidence was unexpectedly high, there have been no attempts to compare renal and other events between statins in Japanese population.

We examined the association between statins and multiple events (increase of creatinine phosphokinase (CK), rhabdomyolysis, increase of aspartate aminotransferase (AST), alanine aminotransferase (ALT), proteinuria, hematuria and increase of serum creatinine) using a prospective stratified case-cohort design. A case-cohort design [12], [13] has both efficiency, an advantage of a case-control study and capability of examining a variety of events, an advantage of a cohort study.

## Methods

### Study Population

Methods complied with the Strengthening the Reporting of Observational Studies in Epidemiology (STROBE) statement [14]. We sent a letter of invitation to join the study to a total of 2,037 hospitals (randomly selected from about 4,000 hospitals with 150 or more beds in the nation) and 68 were enrolled. We identified patients with hyperlipidemia who newly (after 6-months of non-use) started a statin in 68 study hospitals in Japan. Currently, 6 statins (pravastatin, atorvastatin, fluvastatin, pitavastatin, rosuvastatin and simvastatin) are available in Japan and it was in 2005 that rosuvastatin was marketed as the 6th statin in Japan. In the study, first, pharmacists in each hospital identified all patients (prevalent users) who used a statin at least once during some time window using electronic prescription data maintained inside each hospital. The time window was normally a 3-month period but could be longer or shorter and each hospital was allowed to select any time window provided that it was included in a study period between January 1, 2008 and July 31, 2010. Second, to identify new users, patients were excluded if prescribed the same statin in a prior 6-month period. To know whether the patient was prescribed the same statin, we examined both of the electronic prescription record and electronic/non-electronic medical record. The latter record was used to exclude patients who already started the same statin in a different hospital or clinic before the patient was referred to the study hospital. If the patient did not use the same statin but used a different statin or other lipid-lowering drugs during the preceding 6-month period, the patient was included in the final cohort as a “switcher” from another lipid-lowering drug. Finally, pharmacists sent a list of patients anonymized by the study ID number as well as the information on gender, age, generic name of statin and the date when the patient used the statin first to the research office (NPO Drug Safety Research Unit Japan, Tokyo, Japan).

### Sampling of Subcohort

When the study office received a list of new users from each study hospital, 5% of patients in the hospital were randomly selected as members of the random sample subcohort. Furthermore, when no patient was found to be selected as a subcohort member by random sampling in one or more of subgroups subdivided by statin in each hospital (defined as a “missing stratum”), one additional patient was selected (by random sampling) from each missing stratum as an additional subcohort member. The final stratified sample subcohort consisted of random sample subcohort members plus additional subcohort members selected from “missing stratum” in each hospital. This way of additional sampling would selectively increase the sampling fraction from small strata with relatively small additional cost. We thought that this feature would be beneficial particularly when one of small groups needed to be examined to know whether the group had any distinct characteristics while the amount of data obtained from random sample subcohort alone would be felt to be too small.

### Two-stage Data Collection Using Standard Report Forms

We used two types of standard report forms for collecting medical records of cohort members. The data was extracted from electronic or non-electronic medical records. The first simple form was to obtain the data on all cohort members and ask whether or not the patient had blood test for serum CK, AST, ALT and creatinine and urine test for hematuria and proteinuria during the 3-month follow-up period. We also asked whether any of post-dose laboratory test results met the criteria of the increase of CK (>10 upper limit of normal, ULN), AST (>3 ULN), ALT (>3 ULN) or serum creatinine (>1.5 mg/dL) as well as hematuria (+) or proteinuria (++) during the follow-up period. Potential cases were defined as those who met one of these criteria irrespective of the baseline laboratory test results. The second form was sent to obtain the information on all of potential cases and subcohort members pre-selected by the random and additional samplings. The second form was to ask diagnoses of and drugs for co-morbidities including hypertension, diabetes, heart diseases, liver diseases and renal diseases at baseline. We also asked whether or not the patient was a “switcher” from another lipid-lowering drug and the date when the patient stopped the drug during the observation period and date when the patient was lost to follow-up. Specifically for potential cases, we collected the data on the clinical course (narrative description and detailed laboratory test results before and during the follow-up period) by a form for each specific kind of adverse event.

### Adjudication of Cases

We summarized the definition of cases in Table 1. To identify cases likely to be caused by a concomitant condition, the event review committee consisting of three specialists in internal medicine reviewed all the possible cases while blinded for statin used by the patient. A patient was adjudicated as a final case when the definition in Table 1 was met and the statin was judged to be a certain, probable or potential cause [15]. Patients were excluded from the final analysis when a concomitant condition, but not the statin, was judged to be a certain or probable cause.

**Table 1:** Definition of adverse events.

Type of event	Baseline	Post-dose
Renal event		
Increase in serum creatinine*		
	<4 mg/dL	>1.5 mg/dL and ≥1.5-fold increase from baseline value
	≥4 mg/dL	increase of ≥0.5 mg/dL
Hematuria†		increase of 2 or more units from baseline on the scale (−,±, +, ++, +++, ++++)
Proteinuria†		at least ++ following increase of 2 or more units from baseline on the scale (−,±,+,++,+++,++++)
Liver event		
Increase in AST or ALT‡		
	Normal	>3 ULN as a result of increase
	Abnormal	>3 ULN and ≥2-fold increase when baseline was above the ULN
Muscle event		
Increase in CK§	Normal	>10 ULN
Rhabdomyolysis§		>10 ULN of CK and clinical course/symptoms

Abbreviations: ULN, upper limit of normal; AST, aspartate aminotransferase; ALT, alanine aminotransferase; CK, creatinine phosphokinase.

*Modified from Bellomo et al. 2004 [30].

† Criteria used in Klepper MJ and Covert B. 2010 [31].

‡ Criteria used in Guidance of FDA. 2009 [32]. Increase of one or both of AST and ALT was counted as one event.

§ Criteria used in Pasternak RC et al. 2002 [33].

### Sample Size

To calculate the sample size, we used 0.1% as the lowest incidence proportion of AST, ALT and CPK in statin users reported in literature [16]. A sample size of the entire cohort consisting of two groups of the same size was estimated to be 10,436 assuming that the incidence proportion was 0.1% in one group and 0.4% or more in another group when α = 0.05, power = 0.8 and m = 5 (m was the ratio of subcohort to the expected number of cases) and the minimum number of subcohort was estimated to be 130, a little larger than 1% of the entire cohort [17]. As 6 statins were available in Japan, a required total size of the entire cohort was estimated to be 18,000 to 28,000 to make comparisons with enough power between the 3 largest subgroups when those 3 subgroups consisted of 60 to 90% of all patients. We selected 5% of the entire cohort rather than 1% as the random sample subcohort because the size of subcohort could be substantially smaller than the number of cases for events with higher incidence proportion (e.g., proteinuria with 2% of the reported incidence proportion) [16]. During the study conduct, it was found to be hard to achieve the size of 18,000 or more of the entire cohort, but, the attainable sample size (around 7,000) was judged to have enough power (around 0.8) to evaluate events with higher incidence proportion without altering the fraction of subcohort (5%).

### Statistical Analysis

For the entire cohort, the distribution of age and gender was estimated for each of 6 statins and the standardised difference [18] was calculated between pravastatin (reference) and other statins for the proportion of patients in the entire cohort whose blood and urine test results were available during the 3-month follow-up period. Standardised differences of less than 0.1 are generally not considered meaningful [18]. For subcohort members, the mean observation period, co-morbidity at baseline and proportion of “switchers” were also estimated.

The hazard ratio of events was estimated by taking pravastatin as a reference. First, the hazard ratio in case-cohort analyses was estimated using a Cox regression model with the weighting method according to Barlow [19]. The weight was the inverse of the sampling fraction of the stratified subcohort or Ni/ni where Ni and ni were the size of the entire cohort and that of the stratified sample subcohort, respectively, of the i-th statin (i = 1, 2, …, 6). According to Barlow [19], the robust variance was used to estimate the 95% confidence interval (CI). To adjust for confounding, the following potential confounders were included in the Cox regression model: age, gender, status of “switcher”, use of high daily dose (more than “usual” daily dose recommended in the package insert) and concomitant diseases (hypertension, diabetes, heart disease, liver disease and renal disease). In addition, as an ad hoc analysis, we also estimated the hazard ratios by using all the available data in the entire cohort according to Breslow [20] assuming that the patient was not lost to follow-up and observed for 91 days for patients who were not a case nor a subcohort member. We did not collect the latter data because the Breslow’s method [20] was published in 2009 after the current study was started while the Barlow’s method [19] (published in 1999) assumed the use of the data for cases and subcohort members only. All the statistical analyses were conducted by SAS (Cary NC, USA) except for the analyses by the Breslow’s method [20] where R software system was used.

### Ethics

The study protocol was approved by the ethics review committee of the Public Health Research Foundation (Tokyo, Japan) in April 2007 (No.7C0011). By the ethics committee, the waiver of informed consent from individual patients in each study hospital was approved. We used anonymized data with serial study IDs created by the research office.

## Results

A total of 116,418 prevalent users of any statin were identified in the time window set between January 1, 2008 and July 31, 2010 by pharmacists in 68 hospitals. The distribution of 6 statins among prevalent users were roughly the same as that of 6 statins marketed in Japan in 2008 estimated from the sales amounts, drug prices (the price is determined by the government and therefore the same throughout the nation) and the “usual” daily dose given in the package insert. Of the prevalent users, 6,877 (6%) were identified as new users of statins. Table 2 shows data on demographics and other characteristics of the entire cohort and subcohort. Rosuvastatin had the size (n = 2,050) 14 times larger than simvastatin (n = 145) in entire cohort. The stratified sample subcohort consisted of 8.0% of the entire cohort (551/6,877) while the random sample subcohort was 5.1% of the entire cohort (348/6,877). The sampling fraction of subcohort was 19.3% with simvastatin and 6.9% with rosuvastatin indicating that the way of additional sampling in the current study selectively increased the sampling fraction from small strata. The proportion of users of rosuvastatin, atorvastatin and pravastatin was 30%, 26%, and 21%, respectively of the entire cohort while that of pitavastatin, fluvastatin and simvastatin was less than 20%. About 80 (58−90)% of patients had blood test while about 45 (40−53)% of patients had urine test at least once during the 3-month follow-up period. The standardised difference of the fraction of patients who had blood test was −0.13 to 0.07 between pravastatin and the other statins except for that between pravastatin and simvastatin (0.33). The standardised difference was −0.09 to 0.07 for urine test except for that between pravastatin and pitavastatin (−0.20). No patient used the daily dose more than the approved maximum dose a day. It was 3% or less of patients that used the high daily dose (higher than the “usual” daily dose recommended in the package insert) except for simvastatin where 11% of patients used the high daily dose. The proportion of “switchers” was 16% in subcohort or 84% were new users who started a statin after 6-month period of non-use of any lipid-lowering drugs. The proportion of comorbidity in the subcohort was 32% for diabetes, 57% for hypertension, 39% for heart disease, and 14% for renal disease.

**Table 2:** Characteristics of study population.

Characteristics	Pravastatin	Atorvastatin	Fluvastatin	Pitavastatin	Rosuvastatin	Simvastatin	Total
Entire cohort	1,418	1,790	365	1,109	2,050	145	6,877
Median age (range)	67 (11−99)	65 (10−96)	67 (25−99)	67 (18−95)	65 (15−101)	67 (9−89)	66 (9−101)
Fraction of patients with laboratory test in 3 months after statin start (%)							
CK	68	64	72	71	74*	58*	69
AST/ALT	86	84	87	89	90*	74*	87
Serum creatinine	86	81	85	86	87	72*	84
Proteinuria/hematuria	44	42	46	53*	48*	40	46
Subcohort							
Number (% of entire cohort)	116 (8)	124 (7)	48 (13)	94 (8)	141 (7)	28 (19)	551 (8)
Random sample (%)	79 (68)	88 (71)	22 (46)	46 (49)	106 (75)	7 (25)	348 (63)
Additional sample (%)	37 (32)	36 (29)	26 (54)	48 (51)	35 (25)	21 (75)	203 (37)
Male (%)	60 (52)	71 (57)	22 (46)	48 (51)	80 (57)	9 (32)	290 (53)
Median age (range)	67 (22−94)	62 (12−91)	69 (29−89)	68 (31−90)	64 (21−93)	68 (40−88)	65 (12−94)
Mean observation days	78.8	81.1	83.8	80.7	74.9	86.8	79.5
High daily dose†							
Number (%)	2 (2)	0 (0)	1 (2)	3 (3)	1 (1)	3 (11)	10 (2)
Previous lipid-lowering drug							
None (%)	98 (84)	98 (80)	34 (70)	69 (74)	90 (64)	17 (61)	406 (74)
Other statin (%)	11 (10)	13 (10)	7 (15)	18 (19)	36 (25)	5 (18)	90 (16)
Non-statin (%)	7 (6)	13 (10)	7 (15)	7 (7)	15 (11)	6 (21)	55 (10)
Comorbidity at baseline							
Diabetes (%)	35 (30)	35 (28)	14 (29)	32 (34)	59 (42)	4 (14)	179 (32)
Hypertension (%)	68 (57)	62 (50)	25 (52)	59 (63)	84 (60)	18 (64)	316 (57)
Heart disease (%)	46 (40)	39 (31)	18 (38)	42 (45)	61 (43)	9 (32)	215 (39)
Liver disease (%)	8 (7)	11 (9)	8 (17)	8 (9)	13 (9)	4 (14)	52 (9)
Renal disease (%)	15 (13)	16 (13)	5 (10)	15 (16)	21 (15)	4 (14)	76 (14)

Abbreviations: AST, aspartate aminotransferase; ALT, alanine aminotransferase; CK, creatinine kinase.

*Absolute value of standardised difference >0.1.

† High daily dose (higher than the “usual” daily dose recommended in the package insert).

In Table 3, the results of adjudication of events of interest are shown. Of a total of 1,816 possible cases, 1,439 were excluded because they did not meet the criteria in Table 1. For 158 of the remaining 377, the event was judged to be likely caused by concomitant disease, concurrent drug, surgical operation, insertion of an indwelling urethral catheter or other procedure/factor and the 219 cases finally adjudicated by the review committee were analyzed by the Cox proportional hazards model.

**Table 3:** Adjudication results of events of interest.

Classification	Renal event			Liver event	Muscle event	
	Increase inserum creatinine	Hematuria	Proteinuria	Increase inAST or ALT	Increase inCK	Rhabdomyolysis
Potential cases*	351	661	387	343	63	11
Definition of adverse events met†						
No	271	539	358	204	57	10
Yes	80	122	29	139	6	1
Alternativecause likely‡	22	39	8	85	4	0
Alternativecause (number)	Renal disease (7),Surgical operation (6),Other drug (2),Urinary tract/bladderdisease (2), Others (5)	Indwelling urethralcatheter§ (16),Urinary tract/bladderdisease (11),Surgical operation(4), Others (8)	Urinary tract/bladderdisease (3),Other drug (2),Surgicaloperation (1),Others (2)	Surgicaloperation(29), Myocardialinfarction (21),Other drug(13),Others (22)	Myocardialinfarction (3),Surgicaloperation (1)	−
Adjudicated case	58	83	21	54	2	1

Abbreviations: AST, aspartate aminotransferase; ALT, alanine aminotransferase; CK, creatinine phosphokinase.

*Potential cases were those with abnormal post-dose laboratory test results regardless of the baseline level.

† Definition for baseline and post-dose laboratory test results in Table 1.

‡ Alternative cause was evaluated for possible cases with adverse events that met definition in Table 1 by the review committee.

§ Insertion of an indwelling urethral catheter.

Table 4 shows the number of adjudicated cases and the unadjusted and adjusted hazard ratios for the increase of serum creatinine, hematuria, proteinuria and increase in AST/ALT. When pravastatin was used as a reference drug, the adjusted hazard ratio of the increase of serum creatinine was between 2.4 and 2.7 with atorvastatin and fluvastatin but the 95% CI was wide. Otherwise no difference was found between pravastatin and other statins. The Breslow’s method gave estimates similar to those by the Barlow’s method. As a sensitivity analysis, we analyzed data by excluding 16% of “switchers” but the results were essentially the same as those in Table 4.

**Table 4:** Association between statin and events estimated by the case-cohort analysis using the stratified sample subcohort.

Event*	Pravastatin	Atorvastatin	Fluvastatin	Pitavastatin	Rosuvastatin	Simvastatin
Increase in serum creatinine						
Number	11	20	7	7	13	0
Unadjusted HR (95% CI)	reference	1.60 (0.72−3.55)	2.28 (0.79−6.59)	1.05 (0.38−2.90)	0.93 (0.39−2.19)	−
Adjusted† HR (95% CI)						
Barlow’s method	reference	2.74 (0.95−7.89)	2.70 (0.66−10.98)	0.72 (0.21−2.43)	0.99 (0.32−3.08)	−
Breslow’s method‡	reference	2.38 (0.92−6.17)	2.68 (0.73−9.79)	0.71 (0.22−2.29)	0.84 (0.27−2.58)	−
Hematuria						
Number	18	12	4	20	28	1
Unadjusted HR (95% CI)	reference	0.57 (0.26−1.27)	0.79 (0.24−2.60)	1.85 (0.88−3.87)	1.22 (0.63−2.39)	0.62 (0.07−5.43)
Adjusted† HR (95% CI)						
Barlow’s method	reference	0.59 (0.25−1.42)	0.71 (0.20−2.49)	1.16 (0.53−2.52)	1.06 (0.51−2.20)	0.51 (0.05−5.04)
Breslow’s method‡	reference	0.57 (0.25−1.32)	0.69 (0.21−2.29)	1.13 (0.55−2.32)	1.02 (0.51−2.04)	0.48 (0.05−4.40)
Proteinuria						
Number	6	2	1	5	7	0
Unadjusted HR (95% CI)	reference	0.34 (0.06−1.81)	0.73 (0.08−6.58)	1.96 (0.57−6.78)	1.07 (0.33−3.52)	−
Adjusted† HR (95% CI)						
Barlow’s method	reference	0.35 (0.06−1.95)	0.76 (0.09−6.83)	1.43 (0.41−5.03)	0.85 (0.26−2.79)	−
Breslow’s method‡	reference	0.37 (0.07−2.03)	0.81 (0.09−7.09)	1.35 (0.40−4.52)	0.98 (0.30−3.15)	−
Increase in AST or ALT						
Number	8	15	4	8	19	0
Unadjusted HR (95% CI)	reference	1.64 (0.66−4.09)	1.79 (0.49−6.51)	1.65 (0.58−4.69)	1.86 (0.77−4.48)	−
Adjusted† HR (95% CI)						
Barlow’s method	reference	1.69 (0.65−4.38)	1.94 (0.50−7.55)	1.16 (0.40−3.39)	1.96 (0.73−5.23)	−
Breslow’s method‡	reference	1.75 (0.69−4.39)	2.07 (0.54−7.92)	1.22 (0.43−3.49)	1.94 (0.76−4.96)	−

Abbreviations: HR, hazard ratio; CI, confidence interval; AST, aspartate aminotransferase; ALT, alanine aminotransferase.

*Definition of event is given in Table 1.

† Adjusted for age, male, switcher from other lipid-lowering drug, use of high daily dose, hypertension, diabetes, heart disease, liver disease and renal disease.

‡ Estimates using “standard weights” in ref 20 are shown.

A 72-year-old man who newly started atorvastatin soon after hospitalized for stroke had the increased serum CK from 214 IU/L measured on admission (normal range 56−244 IU/L) to 12,954 IU/L on day 3. The patient used no preceding lipid-lowering drugs and had no history of cardiovascular disease. The patient also had edaravone and argatroban for the treatment of stroke. Atorvastatin and other drugs were discontinued and the serum CK level was gradually decreased to 175 IU/L in two months. The review committee determined that the patient was likely to have developed rhabdomyolysis due to atorvastatin. Another 63-years old female patient who had pitavastatin developed the increase in serum CK from 364 to 1,724 (normal range 45−163) IU/L in day 29. Except for these two, no patients had the increase in serum CK as defined in Table 1.

## Discussion

We examined the association between multiple events and a variety of statins by the prospective stratified case-cohort design. The use of statin had no relationship to the increased risk of our targeted events. The adjusted hazard ratio of the increase of serum creatinine for atorvastatin and fluvastatin was, however, around 2.5 when pravastatin was used as a reference drug (Table 4) though the 95% CI was wide and inconclusive.

During the last decade, results of several studies on the renal toxicity associated with the use of statin have been published [6−8], . In pooled analysis of 30 clinical trials, fluvastatin was reported to be safe and effective in chronic renal disease [21]. For atorvastatin, a beneficial effect on renal function was reported in patients with diabetes [22] or coronary heart disease [23]. On the other hand, in a study using the UK database, the risk for acute renal failure was increased with pravastatin and atorvastatin in males and females and fluvastatin in females, compared to no statin users [6]. In another study using the Taiwan National Health Insurance claims database, the renal risk of atorvastatin and rosuvastatin was increased compared to other statins (lovastatin, simvastatin, pravastatin and fluvastatin) [7]. In a recent study on more than 2 million new users of statins using several databases in North America and UK, the use of rosuvastatin, atorvastatin and simvastatin was associated with the increased risk of hospitalization for acute kidney injury compared with other statins [8]. Our finding, together with these studies, may be regarded as a weak alert for the renal toxicity of atorvastatin and fluvastatin.

Two recent meta-analyses [24], [25] suggested that the risk of transaminase increase in pravastatin was lower than that in other statins. Our findings are not contrary to the results but no firm conclusion is possible because 95% CI was wide.

Muscle-related adverse events (rhabdomyolysis, increase of CK, myositis, myalgia and myopathy) [26], [27] are of the several safety concerns associated with statin use. In our study, only rhabdomyolysis and CK increase were included as muscle-related events of interest. Our results suggested that muscle-related adverse events were rare as only two cases were adjudicated to have developed CK increase including one case of rhabdomyolysis in a total of about 7,000 statin new users.

To address the concern of safety of drugs, the primary data collection still has its own role. For instance, claims database generally does not include laboratory test results. It is worth examining an efficient study design like the case-cohort design to broaden options for the studies employable to address a wide range of drug-related problems. In particular, Japanese regulatory authority has often required drug companies to conduct the DUI, a stereotyped cohort study without a comparator group involving primary data collection following the registry of the users of a newly approved drug. The DUI is still prevailing in Japan without thoughtful consideration on whether the study with a comparator is a better option. We believe that the case-cohort design can be one of the future options to improve the company-sponsored post-authorization studies.

To reduce the bias and enhance the efficiency, our study was elaborated in several points. First, to find new users of statins in study hospitals, we used a time window set by pharmacists in the study hospital. New users were then selected from those patients identified in this time window by excluding those who used the same statin in a preceding 6-month period. This way to identify new users was feasible without any special computational skill using the electronic prescription data. Second, we tried to improve the method of subcohort sampling. We expected that the size of the subcohort selected by a simple random sampling could be very small for small subgroups when subdivided by the statin (like a subgroup with simvastatin where only 7 patients were selected as random sample subcohort members as in Table 2). This might create a problem when a small subgroup should be evaluated with a special attention. For instance, in a hypothetical situation, the incidence of a certain event may be found unexpectedly high in a small subgroup (though this did not happen in the current study). We tried a strategy of “one additional subcohort member from a missing stratum in each hospital” and at the end of the study, the proportion of a stratified sample subcohort in the entire cohort was larger for smaller subgroups. It has been suggested that a case-cohort design is subject to a certain type of the information bias [28]. In a case-cohort study, detailed data may be collected from subcohort members early in the study while data from cases arising outside the subcohort can be collected only after the occurrence of an outcome of interest. This may result in the deterioration of the information collected from cases outside the subcohort. In our study, however, we collected the information from subcohort members and all the cases at the same timing (or at the end of follow-up) to avoid bias. In our study, this did not present any problem as the follow-up period was relatively short (3-month period). Special attention and elaboration of data collection methods may be needed in a case-cohort study with a long follow-up period.

In our analysis, estimates by the Breslow’s method to use all of the available data in the entire cohort [20] did not substantially different from those by the Barlow’s method using the data of cases and subcohort only [19]. This was probably because we obtained minimum amount of information except for the cases and subcohort members.

This study has some limitations. New statin use in this study might not be perfectly representative of statin use in entire country. However, the distribution of prevalent users of statin in 68 hospitals was almost the same with that in the whole nation [29]. Second, pediatric patients (<18 years old) were included in this study and the study population may not be homogenous. However, the number of pediatric patients was 10 (0.1%) in entire cohort (n = 6,877) and 2 (0.4%) in subcohort (n = 551) and the effect of the inclusion of pediatric patients on the results would be minimal. Third, during the 3-month follow-up period, the proportion of those who had blood test (around 80%) and urine test (46%) was less than 100% (Table 2) though the proportion was similar between statins. Fourth, some events associated with a drug may occur late and the follow-up period longer than 3-month may be needed to know the occurrence of such events. However, most of renal, liver and muscle events (87% = 854/978) had occurred within the 12-week observation period in a DUI conducted in Japan [11]. For short-term outcomes like in our study, the 3-month follow-up may be therefore adequate. Fifth, this study had insufficient statistical power to detect the association between use of statin and renal, liver and muscle events. The sample size was smaller than initially planned and the 95% CI of the hazard ratio was wide for most events though all the cases were carefully adjudicated by the event review committee. Lastly, the inclusion of the “switchers” in the study population may have impeded the estimation of the correct estimates of the risk as the “switchers” may be regarded as a non-new user of a drug class of lipid-lowering drugs. However, the proportion of the “switchers” was relatively small (16%) and the results were essentially the same even when excluding the “switchers” in the analysis.

## Conclusion

In a prospective case-cohort study involving primary data collection, we found that use of statin was not associated with a significant increased risk of renal, liver and muscle events. However, a weak alert for renal toxicity of atorvastatin and fluvastatin shown in our study require further investigations as our study did not have enough power. As a case-cohort study can examine the association between a variety of outcomes and exposures, the design may be useful in post-marketing studies for newly marketed medicines. In addition, the design may be useful when comparing the subgroups of different size which is often the case in the observational study comparing users of drugs in one class or drugs with the same indication.

## Supporting Information

Checklist S1 **STROBE checklist.**
(DOC)
